# 1-(2-Ureidoeth­yl)quinolinium tetra­phenyl­borate

**DOI:** 10.1107/S1600536811027218

**Published:** 2011-07-13

**Authors:** Zhi-Yun Dong, Yu Yang, Guo-Hua Gao

**Affiliations:** a Shanghai Key Laboratory of Green Chemistry and Chemical Processes, Department of Chemistry, East China Normal University, Shanghai 200062, People’s Republic of China

## Abstract

In the cation of the title salt, C_12_H_14_N_3_O^+^·C_24_H_20_B^−^, the dihedral angle between the quinoline ring and the mean plane of the urea fragment is 61.51 (5)°. In the crystal, the cations inter­act through weak C—H⋯O hydrogen bonding, forming a zigzag chain along the *c*-axis direction; the cations and anions are involved in weak inter­molecular C—H⋯π and N—H⋯π inter­actions as donors and acceptors, respectively.

## Related literature

For applications of ionic liquids, see: Zhao & Malhotra (2002 ▶); Chauvin & Olivier-Bourbigou (1995 ▶); Seddon (2001 ▶); Hapiot & Lagros (2008 ▶); Blaster & Studer (2003 ▶). For a related structure, see: Youngme *et al.* (2006 ▶).
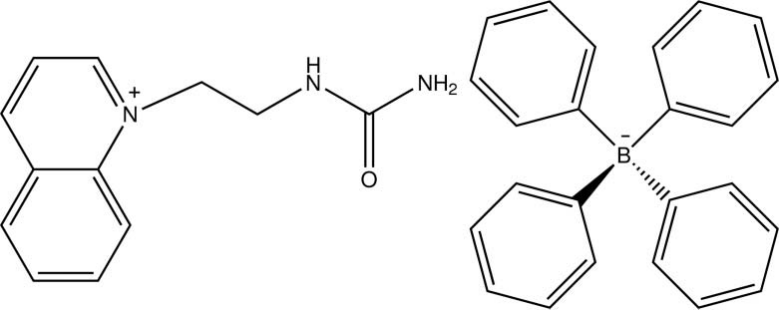


## Experimental

### 

#### Crystal data

C_12_H_14_N_3_O^+^·C_24_H_20_B^−^
*M* *_r_* = 535.47
Orthorhombic, 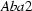

*a* = 25.5434 (11) Å
*b* = 18.7954 (8) Å
*c* = 12.0837 (5) Å
*V* = 5801.4 (4) Å^3^
*Z* = 8
Mo *K*α radiation
μ = 0.07 mm^−1^
*T* = 173 K
0.40 × 0.18 × 0.13 mm

#### Data collection

Rigaku Saturn CCD diffractometer
32920 measured reflections
2700 independent reflections
2428 reflections with *I* > 2σ(*I*)
*R*_int_ = 0.039

#### Refinement

*R*[*F* ^2^ > 2σ(*F* ^2^)] = 0.027
*wR*(*F* ^2^) = 0.070
*S* = 1.06
2700 reflections
370 parameters
1 restraint
H-atom parameters constrained
Δρ_max_ = 0.17 e Å^−3^
Δρ_min_ = −0.13 e Å^−3^

### 

Data collection: *CrystalClear* (Rigaku, 2005 ▶); cell refinement: *CrystalClear*; data reduction: *CrystalClear*; program(s) used to solve structure: *SHELXTL* (Sheldrick, 2008 ▶); program(s) used to refine structure: *SHELXTL*; molecular graphics: *SHELXTL*; software used to prepare material for publication: *SHELXTL*.

## Supplementary Material

Crystal structure: contains datablock(s) global, I. DOI: 10.1107/S1600536811027218/xu5255sup1.cif

Structure factors: contains datablock(s) I. DOI: 10.1107/S1600536811027218/xu5255Isup2.hkl

Supplementary material file. DOI: 10.1107/S1600536811027218/xu5255Isup3.cml

Additional supplementary materials:  crystallographic information; 3D view; checkCIF report

## Figures and Tables

**Table 1:** Hydrogen-bond geometry (Å, °) *Cg*1 and *Cg*2 are the centroids of the C1–C6 and C7–C12 rings, respectively.

*D*—H⋯*A*	*D*—H	H⋯*A*	*D*⋯*A*	*D*—H⋯*A*
C30—H30*A*⋯O1^i^	0.95	2.30	3.173 (3)	153
N2—H2*B*⋯*Cg*2^ii^	0.88	2.62	3.483 (2)	168
C26—H26*A*⋯*Cg*1^iii^	0.95	2.61	3.438 (3)	146
C27—H27*A*⋯*Cg*2^iv^	0.95	2.68	3.572 (3)	157

Symmetry codes: (i) 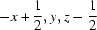; (ii) 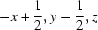; (iii) 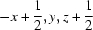; (iv) 
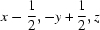
.

**Fig. 1.**
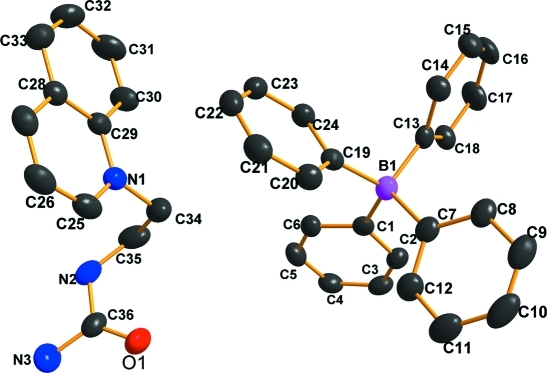
Molecular structure of the title compound, with the atom-numbering scheme. Displacement ellipsoids are drawn at the 50%.

**Fig. 2.**
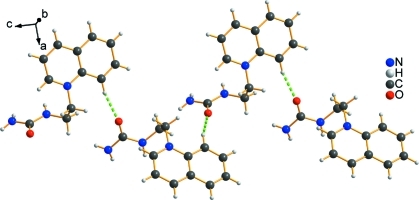
A view of the H-bonds between cations.

**Fig. 3.**
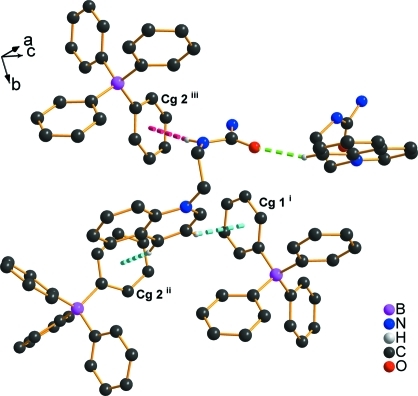
A detailed view of the interactions between cations and anions involves H-bonds, C—H···π and N—H···π, some H atoms have been omitted for clarity.

## supplementary crystallographic information

### Comment

Ionic liquids (ILs) are widely used as new and designable green chemical materials in organic, organometallic and enzymatic catalyses as solvents (Zhao & Malhotra, 2002; Chauvin & Olivier-Bourbigou, 1995; Seddon, 2001) and nano- and electrochemistry (Hapiot & Lagros, 2008; Blaster & Studer, 2003).

In the title salt (Fig. 1), the 1-(2-ureidoethyl)quinolinium cation consists of urea and quinoline ring connected to each other *via* ethylidene. The dihedral angle between the quinoline ring and mean plane of urea is 61.51 (5)°. The 1-(2-ureidoethyl)quinolinium cations interact through a weak C—H···O interaction, forming a zidzag chain along the *C* direction (Fig. 2). The 1-(2-ureidoethyl)quinolinium cation is situated between three [BPh_4_]^-^ anions and this orientation is further stabilized *via* C—H···π and N—H···π interaction (Table 1 and Fig. 3), similar to that found in the structure of tetraphenylborate anion [BPh_4_]^-^ (Youngme *et al.*, 2006).

### Experimental

All chemicals were obtained from commercial sources and used without further purification. A solution of quinoline (0.65 g, 5 mmol) and 2-chloroethylurea (0.61 g, 5 mmol) in ethanol(10 ml) was heated at 353 K with stirring for 72 h under N_2_. After the reaction mixture cooled to room temperature, the purple precipitate was collected and dissolved in water (0.34 g, 40 ml) and treated with a saturated solution of sodium tetraphenylborate (10 ml). The title compound was separated and recrystallized from ethanol. Colourless single crystals were grown from methanol by slow evaporation at ambient room temperature over a period of 18 d.

### Refinement

Positional parameters of all H atoms were calculated geometrically and were allowed to ride on the C atoms to which they are bonded, with *U*_iso_(H) = 1.2*U*_eq_(C). The H atoms bonded to N atoms were refined with a distance restraint of N—H = 0.88 Å.

### Crystal data

**Table:**

C_12_H_14_N_3_O^+^·C_24_H_20_B^−^	*F*(000) = 2272
*M**_r_* = 535.47	*D*_x_ = 1.226 Mg m^−^^3^
Orthorhombic, *A**b**a*2	Mo *K*α radiation, λ = 0.71073 Å
Hall symbol: A 2 -2ac	Cell parameters from 5127 reflections
*a* = 25.5434 (11) Å	θ = 1.6–25.0°
*b* = 18.7954 (8) Å	µ = 0.07 mm^−^^1^
*c* = 12.0837 (5) Å	*T* = 173 K
*V* = 5801.4 (4) Å^3^	Block, colorless
*Z* = 8	0.40 × 0.18 × 0.13 mm

### Data collection

**Table:**

Rigaku Saturn CCD diffractometer	2428 reflections with *I* > 2σ(*I*)
Radiation source: fine-focus sealed tube	*R*_int_ = 0.039
graphite	θ_max_ = 25.0°, θ_min_ = 1.6°
ω scans	*h* = −29→30
32920 measured reflections	*k* = −21→22
2700 independent reflections	*l* = −14→14

### Refinement

**Table:**

Refinement on *F*^2^	Primary atom site location: structure-invariant direct methods
Least-squares matrix: full	Secondary atom site location: difference Fourier map
*R*[*F*^2^ > 2σ(*F*^2^)] = 0.027	Hydrogen site location: inferred from neighbouring sites
*wR*(*F*^2^) = 0.070	H-atom parameters constrained
*S* = 1.06	*w* = 1/[σ^2^(*F*_o_^2^) + (0.0339*P*)^2^ + 1.8667*P*] where *P* = (*F*_o_^2^ + 2*F*_c_^2^)/3
2700 reflections	(Δ/σ)_max_ < 0.001
370 parameters	Δρ_max_ = 0.17 e Å^−^^3^
1 restraint	Δρ_min_ = −0.13 e Å^−^^3^

### Special details

**Table:**

Experimental. ^1^H NMR (Bruker Avance DRX 500 F T NMR spectrometer, DMSO-*d*_6_ solvent, ambient temperature): 3.59 (t, *J* = 5.5 Hz, 2 H), 5.05 (t, *J* = 5.5 Hz, 2H), 5.59 (s, 2 H), 6.16 (s, 1 H), 6.79 (t, *J* = 7.5 Hz, 4 H), 6.92 (t, *J* = 7.5 Hz, 8 H), 7.18 (s, 8H), 8.04 (t, *J* = 7.5 Hz, 1 H), 8.14–8.17 (m, 1 H), 8.27 (t, *J* = 4.5 Hz, 1 H), 8.46 (d, *J* = 8.0 Hz, 1 H), 8.68 (d, *J* = 9.0 Hz, 1 H), 9.26 (d, *J* = 8.5 Hz, 1 H), 9.30 (d, *J* = 5.5 Hz, 1 H).
Geometry. All e.s.d.'s (except the e.s.d. in the dihedral angle between two l.s. planes) are estimated using the full covariance matrix. The cell e.s.d.'s are taken into account individually in the estimation of e.s.d.'s in distances, angles and torsion angles; correlations between e.s.d.'s in cell parameters are only used when they are defined by crystal symmetry. An approximate (isotropic) treatment of cell e.s.d.'s is used for estimating e.s.d.'s involving l.s. planes.
Refinement. Refinement of *F*^2^ against ALL reflections. The weighted *R*-factor *wR* and goodness of fit *S* are based on *F*^2^, conventional *R*-factors *R* are based on *F*, with *F* set to zero for negative *F*^2^. The threshold expression of *F*^2^ > σ(*F*^2^) is used only for calculating *R*-factors(gt) *etc*. and is not relevant to the choice of reflections for refinement. *R*-factors based on *F*^2^ are statistically about twice as large as those based on *F*, and *R*- factors based on ALL data will be even larger.

### Fractional atomic coordinates and isotropic or equivalent isotropic displacement parameters (Å^2^)

**Table:**

	*x*	*y*	*z*	*U*_iso_*/*U*_eq_	
B1	0.36506 (9)	0.28381 (12)	0.1753 (2)	0.0305 (5)	
N3	0.19822 (8)	−0.07125 (10)	0.54551 (19)	0.0488 (5)	
H3A	0.2089	−0.0718	0.6147	0.059*	
H3B	0.1739	−0.1013	0.5234	0.059*	
C1	0.38950 (7)	0.20251 (10)	0.18152 (17)	0.0280 (4)	
C2	0.44354 (8)	0.19105 (10)	0.19212 (17)	0.0325 (5)	
H2A	0.4658	0.2313	0.1985	0.039*	
C3	0.46596 (9)	0.12399 (11)	0.19376 (18)	0.0364 (5)	
H3C	0.5029	0.1191	0.1999	0.044*	
C4	0.43460 (9)	0.06419 (11)	0.18648 (19)	0.0367 (5)	
H4A	0.4496	0.0180	0.1879	0.044*	
C5	0.38123 (9)	0.07275 (11)	0.1771 (2)	0.0373 (5)	
H5A	0.3592	0.0322	0.1723	0.045*	
C6	0.35952 (8)	0.14047 (11)	0.17453 (18)	0.0317 (5)	
H6A	0.3226	0.1448	0.1677	0.038*	
C7	0.39097 (8)	0.33034 (11)	0.27733 (18)	0.0314 (5)	
C8	0.40921 (8)	0.40028 (11)	0.2654 (2)	0.0367 (5)	
H8A	0.4063	0.4228	0.1953	0.044*	
C9	0.43147 (9)	0.43786 (13)	0.3533 (2)	0.0471 (6)	
H9A	0.4434	0.4851	0.3418	0.057*	
C10	0.43627 (9)	0.40757 (15)	0.4555 (2)	0.0494 (7)	
H10A	0.4510	0.4337	0.5151	0.059*	
C11	0.41931 (10)	0.33815 (15)	0.4710 (2)	0.0481 (6)	
H11A	0.4230	0.3160	0.5412	0.058*	
C12	0.39687 (9)	0.30122 (12)	0.38328 (19)	0.0402 (5)	
H12A	0.3850	0.2540	0.3958	0.048*	
C13	0.37940 (8)	0.32221 (10)	0.05635 (18)	0.0299 (4)	
C14	0.35195 (9)	0.38272 (11)	0.02078 (19)	0.0369 (5)	
H14A	0.3247	0.4007	0.0663	0.044*	
C15	0.36284 (10)	0.41740 (12)	−0.0777 (2)	0.0437 (6)	
H15A	0.3437	0.4588	−0.0976	0.052*	
C16	0.40143 (10)	0.39196 (13)	−0.1469 (2)	0.0491 (6)	
H16A	0.4085	0.4148	−0.2155	0.059*	
C17	0.42949 (10)	0.33316 (13)	−0.1151 (2)	0.0481 (6)	
H17A	0.4563	0.3151	−0.1619	0.058*	
C18	0.41890 (8)	0.29986 (11)	−0.0149 (2)	0.0363 (5)	
H18A	0.4396	0.2600	0.0059	0.044*	
C19	0.30087 (8)	0.27991 (10)	0.18398 (19)	0.0300 (4)	
C20	0.27153 (8)	0.29676 (11)	0.27789 (19)	0.0360 (5)	
H20A	0.2893	0.3118	0.3428	0.043*	
C21	0.21687 (9)	0.29227 (12)	0.2799 (2)	0.0429 (6)	
H21A	0.1983	0.3047	0.3452	0.051*	
C22	0.18979 (9)	0.27008 (12)	0.1880 (2)	0.0438 (6)	
H22A	0.1527	0.2673	0.1891	0.053*	
C23	0.21741 (9)	0.25180 (12)	0.0939 (2)	0.0412 (6)	
H23A	0.1993	0.2358	0.0299	0.049*	
C24	0.27154 (8)	0.25679 (11)	0.09271 (19)	0.0347 (5)	
H24A	0.2896	0.2439	0.0270	0.042*	
C25	0.13503 (10)	0.11765 (11)	0.38478 (19)	0.0393 (5)	
H25A	0.1572	0.1149	0.4477	0.047*	
C26	0.08270 (10)	0.13228 (11)	0.3999 (2)	0.0426 (6)	
H26A	0.0689	0.1380	0.4723	0.051*	
C27	0.05106 (10)	0.13850 (11)	0.3102 (2)	0.0392 (5)	
H27A	0.0153	0.1512	0.3196	0.047*	
C28	0.07092 (8)	0.12627 (11)	0.20353 (18)	0.0325 (5)	
C29	0.12449 (8)	0.10883 (10)	0.19089 (18)	0.0317 (5)	
C30	0.14452 (10)	0.09501 (13)	0.08501 (19)	0.0427 (6)	
H30A	0.1803	0.0829	0.0756	0.051*	
C31	0.11207 (11)	0.09919 (14)	−0.0041 (2)	0.0529 (7)	
H31A	0.1258	0.0900	−0.0758	0.063*	
C32	0.05902 (11)	0.11660 (15)	0.0070 (2)	0.0524 (7)	
H32A	0.0372	0.1192	−0.0566	0.063*	
C33	0.03888 (10)	0.12971 (13)	0.1085 (2)	0.0448 (6)	
H33A	0.0029	0.1413	0.1159	0.054*	
C34	0.21205 (9)	0.09099 (14)	0.2790 (2)	0.0498 (6)	
H34A	0.2300	0.1128	0.3432	0.060*	
H34B	0.2269	0.1122	0.2109	0.060*	
C35	0.22221 (10)	0.01157 (14)	0.2788 (2)	0.0515 (6)	
H35A	0.2089	−0.0087	0.2086	0.062*	
H35B	0.2605	0.0035	0.2808	0.062*	
C36	0.21909 (8)	−0.02431 (11)	0.4730 (2)	0.0358 (5)	
N1	0.15554 (7)	0.10719 (9)	0.28474 (15)	0.0349 (4)	
N2	0.19846 (7)	−0.02603 (10)	0.36987 (17)	0.0423 (5)	
H2B	0.1698	−0.0508	0.3581	0.051*	
O1	0.25263 (7)	0.01830 (9)	0.49865 (15)	0.0523 (4)	

### Atomic displacement parameters (Å^2^)

**Table:**

	*U*^11^	*U*^22^	*U*^33^	*U*^12^	*U*^13^	*U*^23^
B1	0.0317 (12)	0.0293 (11)	0.0305 (12)	−0.0016 (9)	0.0020 (11)	−0.0061 (10)
N3	0.0510 (12)	0.0388 (11)	0.0567 (13)	−0.0044 (9)	−0.0027 (11)	0.0042 (10)
C1	0.0339 (11)	0.0305 (10)	0.0198 (9)	−0.0010 (8)	−0.0008 (9)	−0.0021 (8)
C2	0.0361 (11)	0.0311 (10)	0.0303 (11)	−0.0021 (9)	−0.0042 (10)	−0.0058 (9)
C3	0.0392 (12)	0.0388 (12)	0.0313 (11)	0.0054 (10)	−0.0085 (10)	−0.0054 (10)
C4	0.0505 (14)	0.0287 (10)	0.0310 (11)	0.0063 (9)	−0.0019 (11)	−0.0008 (9)
C5	0.0472 (13)	0.0281 (10)	0.0367 (12)	−0.0068 (9)	0.0037 (11)	0.0002 (10)
C6	0.0318 (11)	0.0337 (11)	0.0297 (11)	−0.0028 (8)	0.0026 (10)	0.0003 (9)
C7	0.0251 (10)	0.0325 (11)	0.0367 (12)	0.0046 (8)	0.0024 (10)	−0.0071 (10)
C8	0.0295 (11)	0.0322 (11)	0.0485 (14)	0.0030 (9)	−0.0020 (10)	−0.0101 (10)
C9	0.0387 (13)	0.0372 (12)	0.0654 (18)	0.0026 (10)	−0.0062 (12)	−0.0195 (13)
C10	0.0390 (14)	0.0581 (16)	0.0510 (16)	0.0010 (12)	−0.0057 (12)	−0.0307 (13)
C11	0.0438 (14)	0.0663 (17)	0.0341 (12)	0.0024 (12)	0.0000 (11)	−0.0136 (12)
C12	0.0431 (14)	0.0433 (13)	0.0341 (12)	−0.0043 (10)	0.0023 (11)	−0.0084 (10)
C13	0.0302 (11)	0.0258 (10)	0.0337 (11)	−0.0061 (8)	−0.0016 (9)	−0.0035 (8)
C14	0.0380 (12)	0.0328 (11)	0.0398 (12)	0.0009 (9)	0.0024 (11)	−0.0003 (10)
C15	0.0471 (14)	0.0304 (11)	0.0536 (15)	−0.0015 (10)	−0.0040 (12)	0.0072 (10)
C16	0.0521 (15)	0.0429 (14)	0.0524 (16)	−0.0081 (12)	0.0094 (13)	0.0182 (12)
C17	0.0470 (14)	0.0451 (14)	0.0522 (15)	−0.0009 (11)	0.0196 (12)	0.0081 (12)
C18	0.0372 (12)	0.0290 (11)	0.0426 (13)	−0.0033 (9)	0.0052 (11)	0.0027 (10)
C19	0.0347 (11)	0.0245 (9)	0.0309 (10)	0.0006 (8)	−0.0015 (10)	0.0020 (9)
C20	0.0397 (12)	0.0339 (11)	0.0344 (11)	0.0017 (9)	0.0018 (10)	−0.0018 (9)
C21	0.0388 (13)	0.0423 (12)	0.0474 (14)	0.0070 (10)	0.0102 (11)	0.0066 (11)
C22	0.0298 (11)	0.0444 (12)	0.0571 (15)	0.0001 (10)	0.0001 (12)	0.0149 (12)
C23	0.0390 (13)	0.0403 (12)	0.0444 (13)	−0.0063 (10)	−0.0105 (11)	0.0105 (11)
C24	0.0366 (12)	0.0351 (11)	0.0324 (11)	−0.0027 (9)	−0.0002 (10)	0.0023 (9)
C25	0.0558 (15)	0.0320 (11)	0.0302 (12)	−0.0027 (11)	−0.0017 (11)	−0.0008 (9)
C26	0.0628 (17)	0.0329 (12)	0.0321 (12)	0.0024 (11)	0.0103 (12)	−0.0025 (10)
C27	0.0398 (13)	0.0314 (12)	0.0464 (14)	0.0040 (10)	0.0141 (11)	0.0019 (9)
C28	0.0351 (11)	0.0263 (10)	0.0360 (12)	0.0017 (8)	0.0037 (9)	0.0013 (9)
C29	0.0373 (12)	0.0277 (10)	0.0301 (11)	−0.0001 (8)	0.0011 (10)	0.0015 (9)
C30	0.0440 (14)	0.0484 (13)	0.0356 (13)	0.0138 (11)	0.0100 (11)	0.0040 (10)
C31	0.0748 (19)	0.0552 (16)	0.0286 (12)	0.0153 (13)	0.0067 (13)	−0.0004 (11)
C32	0.0581 (17)	0.0593 (16)	0.0399 (14)	0.0076 (13)	−0.0145 (13)	−0.0013 (12)
C33	0.0384 (13)	0.0471 (13)	0.0489 (14)	0.0042 (11)	−0.0052 (11)	0.0010 (12)
C34	0.0319 (12)	0.0661 (16)	0.0513 (16)	−0.0020 (11)	−0.0030 (12)	0.0160 (13)
C35	0.0414 (14)	0.0718 (18)	0.0413 (14)	0.0180 (12)	−0.0050 (11)	−0.0021 (13)
C36	0.0296 (11)	0.0303 (11)	0.0473 (13)	0.0057 (9)	−0.0065 (10)	−0.0056 (10)
N1	0.0359 (10)	0.0342 (9)	0.0345 (10)	−0.0017 (8)	−0.0021 (9)	0.0041 (8)
N2	0.0372 (10)	0.0397 (10)	0.0499 (12)	−0.0010 (8)	−0.0130 (10)	−0.0058 (9)
O1	0.0484 (9)	0.0545 (10)	0.0540 (10)	−0.0165 (9)	−0.0176 (9)	0.0039 (8)

### Geometric parameters (Å, °)

**Table:**

B1—C19	1.645 (3)	C19—C24	1.402 (3)
B1—C1	1.652 (3)	C19—C20	1.396 (3)
B1—C7	1.650 (3)	C20—C21	1.399 (3)
B1—C13	1.649 (3)	C20—H20A	0.9500
N3—C36	1.353 (3)	C21—C22	1.373 (4)
N3—H3A	0.8800	C21—H21A	0.9500
N3—H3B	0.8800	C22—C23	1.382 (4)
C1—C6	1.398 (3)	C22—H22A	0.9500
C1—C2	1.403 (3)	C23—C24	1.386 (3)
C2—C3	1.385 (3)	C23—H23A	0.9500
C2—H2A	0.9500	C24—H24A	0.9500
C3—C4	1.383 (3)	C25—N1	1.332 (3)
C3—H3C	0.9500	C25—C26	1.377 (3)
C4—C5	1.377 (3)	C25—H25A	0.9500
C4—H4A	0.9500	C26—C27	1.357 (3)
C5—C6	1.389 (3)	C26—H26A	0.9500
C5—H5A	0.9500	C27—C28	1.404 (3)
C6—H6A	0.9500	C27—H27A	0.9500
C7—C12	1.400 (3)	C28—C29	1.415 (3)
C7—C8	1.402 (3)	C28—C33	1.411 (3)
C8—C9	1.396 (3)	C29—N1	1.384 (3)
C8—H8A	0.9500	C29—C30	1.402 (3)
C9—C10	1.365 (4)	C30—C31	1.361 (4)
C9—H9A	0.9500	C30—H30A	0.9500
C10—C11	1.388 (4)	C31—C32	1.400 (4)
C10—H10A	0.9500	C31—H31A	0.9500
C11—C12	1.391 (3)	C32—C33	1.353 (4)
C11—H11A	0.9500	C32—H32A	0.9500
C12—H12A	0.9500	C33—H33A	0.9500
C13—C18	1.391 (3)	C34—N1	1.477 (3)
C13—C14	1.404 (3)	C34—C35	1.515 (4)
C14—C15	1.385 (3)	C34—H34A	0.9900
C14—H14A	0.9500	C34—H34B	0.9900
C15—C16	1.378 (4)	C35—N2	1.442 (3)
C15—H15A	0.9500	C35—H35A	0.9900
C16—C17	1.372 (3)	C35—H35B	0.9900
C16—H16A	0.9500	C36—O1	1.213 (3)
C17—C18	1.390 (3)	C36—N2	1.354 (3)
C17—H17A	0.9500	N2—H2B	0.8800
C18—H18A	0.9500		
			
C19—B1—C1	109.42 (16)	C20—C19—B1	125.24 (19)
C19—B1—C7	112.06 (17)	C19—C20—C21	122.4 (2)
C1—B1—C7	107.72 (17)	C19—C20—H20A	118.8
C19—B1—C13	107.26 (17)	C21—C20—H20A	118.8
C1—B1—C13	111.14 (17)	C22—C21—C20	120.5 (2)
C7—B1—C13	109.28 (16)	C22—C21—H21A	119.8
C36—N3—H3A	120.0	C20—C21—H21A	119.8
C36—N3—H3B	120.0	C21—C22—C23	118.9 (2)
H3A—N3—H3B	120.0	C21—C22—H22A	120.5
C6—C1—C2	114.62 (18)	C23—C22—H22A	120.5
C6—C1—B1	124.18 (17)	C22—C23—C24	120.1 (2)
C2—C1—B1	121.19 (17)	C22—C23—H23A	120.0
C3—C2—C1	123.23 (19)	C24—C23—H23A	120.0
C3—C2—H2A	118.4	C19—C24—C23	123.1 (2)
C1—C2—H2A	118.4	C19—C24—H24A	118.4
C2—C3—C4	120.0 (2)	C23—C24—H24A	118.4
C2—C3—H3C	120.0	N1—C25—C26	122.1 (2)
C4—C3—H3C	120.0	N1—C25—H25A	118.9
C5—C4—C3	118.91 (19)	C26—C25—H25A	118.9
C5—C4—H4A	120.5	C27—C26—C25	119.3 (2)
C3—C4—H4A	120.5	C27—C26—H26A	120.3
C4—C5—C6	120.28 (19)	C25—C26—H26A	120.3
C4—C5—H5A	119.9	C26—C27—C28	120.2 (2)
C6—C5—H5A	119.9	C26—C27—H27A	119.9
C1—C6—C5	123.0 (2)	C28—C27—H27A	119.9
C1—C6—H6A	118.5	C29—C28—C27	119.1 (2)
C5—C6—H6A	118.5	C29—C28—C33	118.9 (2)
C12—C7—C8	115.1 (2)	C27—C28—C33	122.0 (2)
C12—C7—B1	121.28 (19)	N1—C29—C30	122.30 (19)
C8—C7—B1	123.6 (2)	N1—C29—C28	118.08 (19)
C9—C8—C7	122.1 (2)	C30—C29—C28	119.6 (2)
C9—C8—H8A	118.9	C31—C30—C29	119.3 (2)
C7—C8—H8A	118.9	C31—C30—H30A	120.4
C8—C9—C10	120.9 (2)	C29—C30—H30A	120.4
C8—C9—H9A	119.6	C30—C31—C32	121.8 (2)
C10—C9—H9A	119.6	C30—C31—H31A	119.1
C11—C10—C9	119.1 (2)	C32—C31—H31A	119.1
C11—C10—H10A	120.4	C33—C32—C31	119.8 (2)
C9—C10—H10A	120.4	C33—C32—H32A	120.1
C10—C11—C12	119.6 (2)	C31—C32—H32A	120.1
C10—C11—H11A	120.2	C32—C33—C28	120.6 (2)
C12—C11—H11A	120.2	C32—C33—H33A	119.7
C11—C12—C7	123.1 (2)	C28—C33—H33A	119.7
C11—C12—H12A	118.4	N1—C34—C35	111.8 (2)
C7—C12—H12A	118.4	N1—C34—H34A	109.3
C18—C13—C14	114.7 (2)	C35—C34—H34A	109.3
C18—C13—B1	124.62 (18)	N1—C34—H34B	109.3
C14—C13—B1	120.70 (18)	C35—C34—H34B	109.3
C15—C14—C13	123.0 (2)	H34A—C34—H34B	107.9
C15—C14—H14A	118.5	N2—C35—C34	114.2 (2)
C13—C14—H14A	118.5	N2—C35—H35A	108.7
C16—C15—C14	120.1 (2)	C34—C35—H35A	108.7
C16—C15—H15A	119.9	N2—C35—H35B	108.7
C14—C15—H15A	119.9	C34—C35—H35B	108.7
C15—C16—C17	118.9 (2)	H35A—C35—H35B	107.6
C15—C16—H16A	120.5	O1—C36—N2	121.7 (2)
C17—C16—H16A	120.5	O1—C36—N3	123.0 (2)
C18—C17—C16	120.3 (2)	N2—C36—N3	115.3 (2)
C18—C17—H17A	119.8	C25—N1—C29	120.98 (19)
C16—C17—H17A	119.8	C25—N1—C34	117.2 (2)
C17—C18—C13	123.0 (2)	C29—N1—C34	121.74 (19)
C17—C18—H18A	118.5	C36—N2—C35	121.83 (19)
C13—C18—H18A	118.5	C36—N2—H2B	119.1
C24—C19—C20	115.00 (19)	C35—N2—H2B	119.1
C24—C19—B1	119.75 (19)		
			
C19—B1—C1—C6	10.8 (3)	C1—B1—C19—C24	−74.9 (2)
C7—B1—C1—C6	132.9 (2)	C7—B1—C19—C24	165.72 (18)
C13—B1—C1—C6	−107.5 (2)	C13—B1—C19—C24	45.8 (2)
C19—B1—C1—C2	−170.64 (19)	C1—B1—C19—C20	103.8 (2)
C7—B1—C1—C2	−48.6 (3)	C7—B1—C19—C20	−15.6 (3)
C13—B1—C1—C2	71.1 (2)	C13—B1—C19—C20	−135.6 (2)
C6—C1—C2—C3	1.0 (3)	C24—C19—C20—C21	−1.3 (3)
B1—C1—C2—C3	−177.6 (2)	B1—C19—C20—C21	−180.0 (2)
C1—C2—C3—C4	−1.0 (3)	C19—C20—C21—C22	0.6 (3)
C2—C3—C4—C5	0.3 (3)	C20—C21—C22—C23	0.4 (3)
C3—C4—C5—C6	0.3 (3)	C21—C22—C23—C24	−0.8 (3)
C2—C1—C6—C5	−0.4 (3)	C20—C19—C24—C23	0.9 (3)
B1—C1—C6—C5	178.2 (2)	B1—C19—C24—C23	179.7 (2)
C4—C5—C6—C1	−0.2 (3)	C22—C23—C24—C19	0.0 (3)
C19—B1—C7—C12	78.1 (3)	N1—C25—C26—C27	−2.1 (3)
C1—B1—C7—C12	−42.3 (3)	C25—C26—C27—C28	3.5 (3)
C13—B1—C7—C12	−163.18 (19)	C26—C27—C28—C29	−1.3 (3)
C19—B1—C7—C8	−102.9 (2)	C26—C27—C28—C33	177.7 (2)
C1—B1—C7—C8	136.7 (2)	C27—C28—C29—N1	−2.4 (3)
C13—B1—C7—C8	15.9 (3)	C33—C28—C29—N1	178.66 (19)
C12—C7—C8—C9	−0.1 (3)	C27—C28—C29—C30	178.6 (2)
B1—C7—C8—C9	−179.3 (2)	C33—C28—C29—C30	−0.3 (3)
C7—C8—C9—C10	−0.2 (4)	N1—C29—C30—C31	−178.4 (2)
C8—C9—C10—C11	0.9 (4)	C28—C29—C30—C31	0.5 (3)
C9—C10—C11—C12	−1.3 (4)	C29—C30—C31—C32	−0.3 (4)
C10—C11—C12—C7	1.0 (4)	C30—C31—C32—C33	−0.1 (4)
C8—C7—C12—C11	−0.3 (3)	C31—C32—C33—C28	0.2 (4)
B1—C7—C12—C11	178.9 (2)	C29—C28—C33—C32	0.0 (3)
C19—B1—C13—C18	−137.6 (2)	C27—C28—C33—C32	−179.0 (2)
C1—B1—C13—C18	−18.1 (3)	N1—C34—C35—N2	−54.0 (3)
C7—B1—C13—C18	100.7 (2)	C26—C25—N1—C29	−1.7 (3)
C19—B1—C13—C14	43.7 (2)	C26—C25—N1—C34	−179.3 (2)
C1—B1—C13—C14	163.26 (18)	C30—C29—N1—C25	−177.2 (2)
C7—B1—C13—C14	−78.0 (2)	C28—C29—N1—C25	3.8 (3)
C18—C13—C14—C15	0.5 (3)	C30—C29—N1—C34	0.3 (3)
B1—C13—C14—C15	179.3 (2)	C28—C29—N1—C34	−178.71 (19)
C13—C14—C15—C16	1.3 (4)	C35—C34—N1—C25	93.1 (3)
C14—C15—C16—C17	−1.6 (4)	C35—C34—N1—C29	−84.5 (3)
C15—C16—C17—C18	0.2 (4)	O1—C36—N2—C35	13.3 (3)
C16—C17—C18—C13	1.7 (4)	N3—C36—N2—C35	−169.2 (2)
C14—C13—C18—C17	−2.0 (3)	C34—C35—N2—C36	−77.0 (3)
B1—C13—C18—C17	179.3 (2)		

### Hydrogen-bond geometry (Å, °)

**Table:**

Cg1 and Cg2 are the centroids of the C1–C6 and C7–C12 rings, respectively.

**Table:**

*D*—H···*A*	*D*—H	H···*A*	*D*···*A*	*D*—H···*A*
C30—H30A···O1^i^	0.95	2.30	3.173 (3)	153
N2—H2B···Cg2^ii^	0.88	2.62	3.483 (2)	168
C26—H26A···Cg1^iii^	0.95	2.61	3.438 (3)	146
C27—H27A···Cg2^iv^	0.95	2.68	3.572 (3)	157

Symmetry codes: (i) −*x*+1/2, *y*, *z*−1/2; (ii) −*x*+1/2, *y*−1/2, *z*; (iii) −*x*+1/2, *y*, *z*+1/2; (iv) *x*−1/2, −*y*+1/2, *z*.

## References

[bb1] Blaster, H. U. & Studer, M. (2003). *Green Chem.* **5**, 112–117.

[bb2] Chauvin, Y. & Olivier-Bourbigou, H. (1995). *Chemtech*, **25**, 26–30.

[bb3] Hapiot, P. & Lagros, C. (2008). *Chem. Rev.* **108**, 2238–2264. 10.1021/cr0680686 18564878

[bb4] Rigaku (2005). *CrystalClear* Rigaku Corporation, Tokyo, Japan.

[bb5] Seddon, K. R. (2001). *Chem. Commun.* **23**, 2399–2407.

[bb6] Sheldrick, G. M. (2008). *Acta Cryst.* A**64**, 112–122. 10.1107/S0108767307043930 18156677

[bb7] Youngme, S., Wannarit, N. & Chaichit, N. (2006). *Acta Cryst.* E**62**, o5265–o5267.

[bb8] Zhao, H. & Malhotra, S. V. (2002). *Aldrichim. Acta*, **35**, 75–83.

